# Progress in Rice Breeding Based on Genomic Research

**DOI:** 10.3390/genes15050564

**Published:** 2024-04-27

**Authors:** Xingye Yang, Shicong Yu, Shen Yan, Hao Wang, Wei Fang, Yanqing Chen, Xiaoding Ma, Longzhi Han

**Affiliations:** 1 State Key Laboratory of Crop Gene Resources and Breeding, Institute of Crop Sciences, Chinese Academy of Agricultural Sciences, Beijing 100081, China; 82101235059@caas.cn (X.Y.); yanshen@caas.cn (S.Y.); 82101221349@caas.cn (H.W.); fangwei@caas.cn (W.F.); chenyanqing@caas.cn (Y.C.); 2 State Key Laboratory of Crop Gene Exploration and Utilization in Southwest China, Rice Research Institute, Sichuan Agricultural University, Chengdu 611130, China; shicongyu@aliyun.com; 3 National Crop Genebank, Institute of Crop Science, Chinese Academy of Agricultural Sciences, Beijing 100081, China

**Keywords:** rice, genome sequencing, resequencing, rice seeding

## Abstract

The role of rice genomics in breeding progress is becoming increasingly important. Deeper research into the rice genome will contribute to the identification and utilization of outstanding functional genes, enriching the diversity and genetic basis of breeding materials and meeting the diverse demands for various improvements. Here, we review the significant contributions of rice genomics research to breeding progress over the last 25 years, discussing the profound impact of genomics on rice genome sequencing, functional gene exploration, and novel breeding methods, and we provide valuable insights for future research and breeding practices.

## 1. Introduction

Rice is one of the most widely grown and essential staple crop species worldwide. As the world’s population has increased, the growth rate in rice production has slowed, resulting in yields that cannot meet the increased quantities of human consumers. According to projections, the global population is expected to reach 9.7 billion by 2050, and global food production may need to increase by more than 70% to meet the world‘s food demand [1]. In addition to climate change, frequent disasters such as drought and high temperatures threaten rice yields and quality; to address these issues, it is imperative to employ quick and efficient genetic improvement strategies. In recent years, progress in rice genomics has been pivotal for advancements in techniques and methods employed in rice genetic improvement. Genomics encompasses structural, functional, epigenetic, and comparative genomics [2,3]. Utilizing genomic information aids breeders in pinpointing crucial gene modules, analyzing the mechanisms underlying essential traits, and providing guidance for genetic improvement [4,5].

Researchers have advanced rice genomics at a high speed in the last century. In 1998, the Rice Genome Research Program entered a new phase of genome sequencing, presenting an excellent opportunity to uncover complete genomic sequence information for rice species [6]. In recent years, the “(3K Rice) Rice Genome Project” has made crucial strides in revealing the genomic diversity of all rice germplasm resources worldwide [7]. The development of genomics-assisted breeding has deepened our understanding of the transmission and permeation of crucial traits and quantitative trait loci (QTLs) within the genetic background of rice. This progress has provided significant assistance in enhancing the rice-breeding process [8]. Furthermore, with the continuous advancement of genomic knowledge and technology, significant progress has been made in research on rice heterosis inheritance and its molecular basis. However, understanding the underlying mechanisms remains challenging, and delving deeper into the mechanism of rice heterosis will contribute to the formulation of new breeding strategies to address the challenges of global food security [9,10]. In rice breeding, genetic variation and gene editing technologies such as mutagenesis and genetic transformation also play significant roles. The application of these technologies can generate new variants of transgenic rice, providing important genetic resources and tools for breeding new varieties with disease resistance, high yield, and stress tolerance. Therefore, the integration of genomics-assisted breeding with genetic variation and gene editing technologies will become crucial components of future breeding strategies in rice [11,12]. In this study, we focused on comparing the contributions of sequencing technologies to rice breeding in different eras. We discussed the progress and prospects of the genomics-based identification of excellent functional genes in rice.

## 2. Rice Genome Sequencing

Genome sequencing can be broadly categorized into three stages: de novo sequencing, resequencing, and large-scale resequencing. Technological advances have addressed the limitations of genome sequencing to varying degrees, consequently providing more accurate and comprehensive genomic information. The high quality and integrity of reference genomes are essential for studies of genome structure, functional gene annotation, genetic variation, and biological evolution. Rice was the first major cereal crop to possess a reference genome. The advent of sequencing technologies has facilitated the generation of numerous high-quality reference genomes and resequencing datasets, which have been extensively applied in genetic research and rice breeding. These have contributed significantly to the substantial development of the rice industry.

### 2.1. De Novo Sequencing

Following the initial appearance of the reference genome, subsequent efforts were primarily focused on the development of a more suitable selection of reference genomes for genetic materials exhibiting significant genetic variations. De novo sequencing involves comprehensive sequencing of the genome of an individual organism without reliance on a known reference genome sequence. The objective is the discovery of novel genes and genomic variations, and this approach is also employed for investigations into the evolution, genetic diversity, and structural and functional aspects of a species genome.

The first reference genome for a crop was generated by the International Rice Genome Sequencing Project (IRGSP). This effort provided a detailed blueprint for all chromosomes of the Nipponbare variety, marking the beginning of crop genomics research. In 2002, the Chinese Academy of Sciences took the lead in sequencing the whole genome of the indica rice variety 9311 and was the first to complete the construction of the indica rice genome framework [13,14]. The completion of the sequencing of the Nipponbare rice genome in 2005 marked the conclusion of the International Rice Genome Sequencing Project, ushering in the era of genomics research with rice as a model crop. Using third-generation sequencing technology, high-quality reference genome sequencing of indica rice was ultimately completed [15]. One study assembled the indica rice genome of the Shuhui498 (R498) rice variety via de novo assembly by integrating single-molecule sequencing, mapping data, genetic mapping, and fosmid sequence tags and annotating high-quality protein-coding genes, which provided a robust strategy for the de novo integration of highly contiguous and nearly complete plant genomes [16].

Recently, researchers have employed a hybrid sequencing strategy that integrates various sequencing technologies for de novo-sequencing purposes. For instance, combining short-read and long-read sequencing enhances genome coverage and accuracy. Liang et al., reported that the indica rice variety Xiangyaxiangzhan (XYXZ) is characterized by excellent phenotypes and quality. They employed Illumina paired-end whole-genome shotgun sequencing and nanopore sequencing technology for de novo assembly and annotation, obtaining high-quality data. Through analysis, they linked specific traits and genetic variations, particularly focusing on the genetic basis of rice quality. This approach facilitates the accelerated breeding of rice varieties of superior quality [17]. These studies are capable of generating high-quality genomic data, which facilitates comprehension of the genetic characteristics and genomic structure of rice. This, in turn, serves as a foundation for subsequent selection of suitable reference genomes. De novo sequencing enables us to acquire more genetic information about rice, and this approach is essential for advancing rice research.

### 2.2. Resequencing

The primary objective of resequencing is to elucidate the distinctions between individuals by examining the genomic levels of diverse materials and furnishing data on pivotal gene loci for elucidating phenotypic variations. Since approximately 2008, with the maturity and application of high-throughput sequencing and nanopore sequencing technologies, research on rice resequencing has gradually increased. High-throughput sequencing technologies (e.g., Illumina, Ion Torrent, etc., San Diego, CA, USA) are not only characterized by high throughput, low cost, and high accuracy but are also suitable for large-scale genome sequencing and population studies, and they are especially good at short sequences. Furthermore, not only do nanopore sequencing technologies (e.g., PacBio, (Menlo Park, CA, USA), Oxford Nanopore (Oxford, UK), etc.) involve long-read sequencing, real-time sequencing, direct sequencing of RNAs, and less bias but they are also useful for solving complex genome structures, detecting repetitive regions of the genome, and supporting real-time analyses and transcriptomics, and they have much more robust performance. Combining allele-specific polymerase chain reaction (AS-PCR) with computational phasing can achieve relatively high throughput. Resequencing of different rice varieties offers a cost-effective, rapid, and efficient means to understand genetic variation in rice, enabling the identification of single nucleotide polymorphisms (SNPs) and insertion/deletion (InDel) variants [18]. The use of these variant details to localize crucial genes and quantitative trait loci (QTLs) is beneficial for exploring functional genomics in rice. This approach aids in uncovering gene functions and regulatory networks and subsequently assists in genetic improvement, accelerating the breeding process.

Studies into diversity among rice varieties from different regions can be conducted using various methods based on different traits and genomic regions [19]. The Rice Bakanae disease (BD), caused by Fusarium fujikuroi, presents a significant threat to rice cultivation areas globally. The discovery of the QTL qFfR9 on rice chromosome nine holds considerable importance for the development of BD-resistant rice varieties. Through genome resequencing of the "Junam" and "Samgwang" varieties, a total of 151,916 DNA polymorphisms were revealed, encompassing single nucleotide polymorphisms (SNPs), insertions/deletions (indels), and structural variations (SVs). A comparison of the sequence data from these two varieties identified eight candidate genes carrying nonsynonymous SNPs, providing valuable insights for identifying the gene associated with qFfR9 [20]. Through the analysis of resequencing data from 33 wild species of rice, different alleles of the haploid induction gene OsCENH3 were identified [21]. Han et al., utilized high-throughput resequencing technology to analyze genes associated with drought tolerance in wild rice, revealing the evolutionary history and selection pressure acting on these genes [22]. Through whole-genome resequencing of four rice NU lines (NU3, NU4, NU5, and NU7) as well as the parental lines *Oryza sativa* WAB56-104 and *Oryza glaberrima* CG14, it was revealed that the NU varieties harbor genes related to biotic stress resistance and pathogenesis [23]. These research studies provide valuable resources for breeding disease-resistant rice varieties.

The process of extensive marker and population analysis in gene identification often requires a significant amount of time and effort, with experimental procedures being intricate, leading to slow progress [24]. Takagi et al., employed the QTL-seq method, used whole-genome resequencing technology to sequence the DNA of two mapping populations rapidly, successfully identified QTLs associated with various traits, such as seedling vigor and disease resistance, and developed an efficient MutMap method. MutMap can accelerate the genetic improvement of rice [25]. Xie et al., utilized resequencing and population genetics analysis to identify 200 genomic regions under selection associated with male sterility and restoration subgroups in a three-line hybrid system [26]. Kumagai et al., validated 50 rice varieties using the program TASUKE and obtained good results [27]. The bulked segregant analysis (BSA) method enables the rapid and precise localization and identification of genes or loci associated with traits of interest [28]. The integration of BSA with pooled DNA whole-genome resequencing enhanced the emergence of high-throughput sequencing technologies.

### 2.3. Large-Scale Resequencing and Pangenomic Methods

The sequencing of a few samples or even dozens of samples does not provide a comprehensive picture of the important selective events in rice evolution. Furthermore, it is difficult for researchers to gain a holistic view of rice cultivation resources. The introduction of large-scale resequencing technology for rice genome research has enabled researchers to study rice as a whole for the first time in a comprehensive way. During the period from 2010 to 2020, there was a notable increase in the number of large-scale resequencing projects. Large-scale resequencing requires the collection of vast amounts of sequence data. Using the Illumina NovaSeq 6000 platform and the R498 genome as a reference, 1143 indica rice germplasms were resequenced, and their genetic variations were analyzed. This analysis revealed various types of single nucleotide polymorphisms (SNPs) and insertion–deletion differences, which were most commonly shared among the two-member and three-member series [29]. By combining NGS and Sanger sequencing, a resequencing effort was conducted for genetic resources of rice from over 1500 diverse populations, enabling the rapid analysis of global rice germplasm diversity [30]. Large-scale resequencing projects in rice have yielded high-quality genome-wide variation information for many different germplasms, opening the possibility of in-depth research on the potential of genome prediction in rice germplasm management and development [31]. This not only enables us to comprehensively understand the genetic diversity of rice but also establishes a robust foundation for optimizing breeding strategies and driving agricultural innovation.

Pangenomic research gradually emerged after 2015, and to better capture genomic variations within populations, researchers began employing pangenomes as references for a more comprehensive understanding of the genetic diversity of rice [32]. In contrast to methods that focus on individual entities, pangenomic research can encompass the entire genetic information of a species or population [33]. The pangenomic study of rice has established the largest and most comprehensively annotated single-genus suprapanome in the field of plant research. Zhao et al., conducted deep sequencing on 66 distinct germplasm- and de novo-assembled-pangenomic datasets for *O. sativa* (cultivated rice) and *O. rufipogon* (wild rice) species complexes. By utilizing the pangenomic dataset, a comprehensive comparison of different assemblies at the whole-genome level was performed, revealing intricate genetic variations [34]. Zhang et al., employed third-generation sequencing (TGS) technology for the construction of a high-quality pangenome in rice. The rice pangenome comprises a total of 879 Mb of new sequences and 19,000 new genes [35]. Through pangenomic analysis of 3010 genomes of Asian cultivated rice (*Oryza sativa* L.) from the “3000 Rice Genomes Project,” more than 10,000 novel full-length-protein-coding genes and presence–absence variations were identified [36]. Wang et al., integrated resequencing datasets from 10,548 cultivated and wild rice varieties to construct a rice superpopulation variation map (RSPVM), demonstrating that using larger population sizes enables a more comprehensive characterization of genomic variation, which is especially important for identifying rare variants [37].

Derivative genomic technologies encompass various aspects, with exon sequencing, simplified genomes, liquid-phase chips, and solid-phase chips being important derivative techniques. Exons are regions in the genome that encode proteins, and there has been a primary focus on sequencing these functional areas. Simplified genomics involves simplifying the genomic structure of an organism by removing or reducing nonessential genes. This approach aids in understanding the relationships between genes and the functionality of the organism. Liquid-phase chips are high-throughput-biological-assay platforms that employ microfluidic technology for bioanalysis in a liquid environment. They can simultaneously process multiple samples, exhibiting the characteristics of high sensitivity and rapid response. These chips are widely utilized in research areas such as gene expression, protein–protein interaction, and cell signaling pathway studies. Solid-phase chips are experimental platforms based on high-density arrangements of DNA, RNA, or protein probes. These methods are utilized for the simultaneous detection of multiple targets. Solid-phase chips enable the rapid and high-throughput analysis of thousands of genes or proteins at the same time, all within a single time point. These derivative technologies promote the rapid utilization of genomes in rice varieties, providing support for the advancement of genetic improvement in rice [38].

## 3. Functional Gene Mining in Rice Genomics Research

Following the sequencing of the rice genome, the mining of key functional genes related to rice yield, quality, disease resistance and stress tolerance has become a significant undertaking that has attracted considerable attention. By cloning the key genes that regulate trait formation, a more comprehensive understanding of the molecular mechanism of important trait formation in rice can be gained, providing a deeper and more comprehensive theoretical basis for practical guidance for rice research.

### 3.1. Genome-Supported Cloning of Functional Genes

The release of the Nipponbare reference genome for rice, among others, has greatly accelerated the pace of functional gene discovery in rice genomics. First, the reference genome provides accurate positional and sequence information for rice genes, facilitating the easier localization and identification of genes associated with specific traits or functions. Second, the use of the reference genome enables the comparison of genomic differences between rice varieties, enabling the identification of shared genes, variations, and functional elements. Furthermore, with the use of a reference genome, researchers can identify potential candidate genes more rapidly. Through gene cloning technology, researchers are able to delve into the key traits related to rice yield and quality, further exploring their functions and regulatory mechanisms, thereby providing a scientific basis for targeted variety improvement. For instance, the discovery of the multi-effect gene DEP1, as well as the low-temperature responsive gene cold1 and high-temperature responsive gene TT1, not only expands our understanding of the growth and development mechanisms of rice but also provides crucial theoretical foundations for variety improvement and yield enhancement.

The identification and mining of genes related to rice growth through genomics contributes to precise breeding. DEP1 has been identified as a beneficial allele gene that enhances rice meristematic tissue activity and increases grain yield, and it is also a key QTL for rice-grain size. A comparison of DEP1 and dep1NIL lines revealed that rice-spike yield was higher in the presence of DEP1. The main role of DEP1 is to regulate the structure of the rice spike, especially the compactness and verticality of the spike. The identification of the DEP1 locus offers the prospect of a deeper understanding of the molecular basis of spike meristemization in rice and provides an avenue for regulating the yield of key crops [39]. The three Gγ proteins, DEP1, GGC2, and GS3, work together to regulate grain size in rice; DEP1 and GGC2, when used alone or in combination, increase grain length in combination with Gβ, whereas GS3 leads to a shortening of grain length through competitive interactions with Gβ. This regulation of the three Gγ proteins allows for the precise design of rice-grain size, thereby enhancing rice yield and quality [40]. Xue et al., identified and successfully cloned a quantitative trait locus (QTL), Ghd7, encoding a protein in the CCT structural domain from a superior rice hybrid that negatively regulates heading onset in rice [41]. With different functional alleles of Ghd7, researchers can adjust the growth period and yield of rice according to various breeding needs [42,43]. Hu et al., reported that Ghd7 suppressed seed dormancy by modulating the balance of ABA and GA, confirming that Ghd7 is associated with seed germination [44]. Herath found that the related QTLs of Ghd7 can be modified to develop superior rice varieties suited to the climate [45]. GW2 is involved in the regulation of rice-grain development, especially in the negative regulation of grain width, and its deletion or mutation may lead to the widening of rice grains [46,47]. The allele gw2.1 of GW2 positively regulates grain shape and is a promising target for improving the grain yield of rice [48].

The OsTT1 gene is involved in regulating the response of rice to high temperatures during its growth and development. Researchers can selectively breed rice varieties with stronger resistance to high-temperature stress by gaining a deeper understanding of the function and regulatory mechanisms of the OsTT1 gene under high-temperature conditions. Li et al., identified thermotolerance 1 (TT1), a gene associated with heat tolerance, as a major quantitative trait locus (QTL) in African rice (*O. glaberrima*) and found that overexpression of OgTT1 was associated with a significant increase in heat tolerance in rice. It was demonstrated that OgTT1 is more effective than OsTT1 in cytoprotection, and that it could more effectively remove toxic denatured proteins produced during heat stress and improve the ability to maintain the heat-stress-response process [49]. COLD1 is one of the key genes involved in the low-temperature response in rice. Its mutation or loss may increase rice’s sensitivity to low temperatures, thereby affecting its growth and development. Using map-based cloning, the important cold tolerance gene COLD1, which is located on chromosome 4, was successfully cloned. Under low-temperature conditions, the COLD1 protein interacts with another protein, RGA, to sense low-temperature signals and transmit them to the nucleus through calcium signaling, thus activating the expression of downstream cold-tolerance-defense genes to allow the plant to withstand chilling stress [50]. These findings lay a solid foundation for future rice genomics research and promote the rice-breeding process, as well as providing strong support for increasing rice yields and stress tolerance.

### 3.2. Functional Gene Mining for Transcriptome Services

Early biological research focused on gene cloning and functional gene studies. While functional gene studies have played an important role in analyzing the regulation of rice growth and development processes, transcriptomics is also needed for the construction of expression regulatory networks. Transcriptomics is suitable for studying plant stress responses, especially non-biological stress and ion absorption regulation. The transcriptome is divided into a reference genome and a nonreference genome, and in rice research, transcriptomes with reference genomes are commonly used. Transcriptome research has gone through stages of development from the use of Northern blotting to the adoption of advanced technologies such as RNA sequencing (RNA-seq), which has revealed genes and their interrelationships that are involved in various developmental stages of plants [51]. Rapid progress in rice breeding has benefited from the development of novel sequencing and genomic technologies, particularly methods such as RNA sequencing (RNA-seq) [52]. Deep sequencing technology has been widely employed in studying the transcriptomes of various organisms to simultaneously quantify and annotate cellular transcripts. Compared to methods such as SAGE, CAGE, and MPSS, RNA-seq offers the advantage of fully covering the transcriptome [53]. The development of these technologies has facilitated a deeper understanding of gene expression levels, revealing the activation or inhibition of genes across different growth stages or specific environmental conditions. Through an in-depth study of the dynamic expression of genes, we can more comprehensively understand the responses of plants to external stimuli and provide more databases for use in plant breeding research.

Transcriptomic research has provided valuable insights for enhancing the resistance, adaptability, and yield of rice and other crops, thereby greatly contributing to global agricultural production. Through comparative analysis of the transcriptome data and gene expression data of rice varieties, Wang et al., found that Gangyuan 8 and Yanfeng 47 infected with *Rhizobium agalactia* AG1-IA demonstrated a defense mechanism against infections by this pathogen [54]. Furthermore, AG1-IA infection can activate a variety of resistance pathways involving different genes involved in defense responses and signaling against pathogen infection. These results suggest that the response mechanism of rice to infection by this pathogen is regulated by a network of multiple genes. Transcriptome studies analyzing the ten most resistant and ten most susceptible individuals demonstrated that overexpression of the WAK91 gene improves rice resistance to rice blast, and through the 3000 Rice Genome Project and publicly available genomic data, it was determined that the resistance genes in the WAK91 SNP mutant appeared to be ancestrally inherited, and it was concluded that the WAK91 gene is a candidate for improved resistance to the leaf blight gene [55]. Under salt–alkali stress conditions, a comparative analysis of the transcriptomes of a salt–alkali-tolerant subspecies (RPY Geng) and a relatively sensitive subspecies (Luohui 9) of rice revealed the specific upregulation of multiple genes associated with salt–alkali tolerance in RPY Geng [56]. To understand how rice responds to various abiotic stresses, the transcriptome of IAC1131 rice related to interactions with these stresses was analyzed after various ABA pretreatments [57]. These studies provide important clues for understanding the underlying mechanisms of rice tolerance to various abiotic stresses. Additionally, these genes are crucial candidate target genes for improving rice resilience.

In addition to transcriptomics, technologies such as single-cell RNA sequencing (scRNA-seq) have also been applied to rice. This technique can reveal differences in gene expression at the single-cell level, enhancing the molecular understanding of cell type differentiation. For instance, Wang et al. characterized the transcriptome of 237,431 single cells using scRNA-seq sequencing, which provided a data resource for the construction of a single-cell transcriptome atlas of rice seedlings by investigating the underlying molecular mechanisms of single cells [58]. Zong et al. utilized single-cell RNA sequencing (scRNA-seq) to analyze rice floral cells, reconstructed the differentiation trajectories of small flowers and meristematic tissue cells, and constructed a single-cell gene expression atlas. Through transcriptomic data, they identified discrete cell types and regulatory factor groups within highly heterogeneous floral meristems [59]. Zhang et al. utilized the obtained single-cell transcriptomic atlas to identify the majority of cell types in rice roots and demonstrated that the root tip of rice is composed of highly heterogeneous cells. By reconstructing the developmental trajectories of cells, they elucidated the regulatory network determining cell fates in the within cell lineages [60]. Utilizing single-cell RNA sequencing (scRNA-seq) technology to identify unknown or rare cell types or states and exploring the cell types or subpopulations present in rice tissues is conducive to elucidating rice root development and holds significant importance for advancing rice genetic improvement.

### 3.3. Functional Gene Mining by Multi-Omics

In addition to genome and transcriptome techniques, other commonly used histological analysis techniques in rice involve the proteome and metabolome. Gene transcription levels and protein content may differ, and proteomics is an important tool for analyzing protein expression, protein interactions, and post-translational modifications of proteins; proteins are the material basis of organisms, but unlike in animals, secondary metabolites in plants are also key substances that constitute plant tissues and regulate physiological processes. Metabolomics research unveils the spectrum of metabolic products in rice, offering crucial information for understanding its adaptive responses and environmental resilience. As the technology becomes more mature, more and more researchers are choosing to explore molecular mechanisms in rice in greater depth using multi-omics analysis [61,62].

The emergence of high-throughput epigenome mapping technologies provides more comprehensive information for multi-omics studies, marking the dawn of a new era in multi-omics research [63]. CARMO is an annotation platform dedicated to the in-depth exploration of rice omics data. This approach comprehensively collects and integrates information from various functional evidence and multi-omics data sources to construct gene sets and higher-level gene modules. CARMO serves as a powerful tool for obtaining a multifaceted understanding of rice omics data [64]. Weighted gene co-expression network analysis (WGCNA) was employed for integrating multi-omics data from different levels. The combined application of WGCNA and multi-omics enables a more comprehensive understanding of the molecular-level interactions and regulatory mechanisms taking place in rice. By constructing co-expression networks, WGCNA reveals the patterns of interactions between molecules [65,66,67]. Genome-wide association studies (GWASs) can analyze extensive genetic variation data, facilitating association analysis across multiple levels of data in multi-omics studies [68]. This has advanced the study of complex traits in rice, particularly in the context of salt tolerance [69,70]. GWASs hold promise for rapidly advancing the rice-breeding process.

Studies of multi-omics interactions have greatly contributed to the understanding of complex biological processes in organisms. For example, by integrating transcriptomic and metabolomic approaches, researchers have revealed the importance of specific genes in regulating the phenylpropane pathway in response to cold stress. Gu et al. demonstrated the crucial role of the OsSEH1 gene in this process [71], while Li et al. elucidated the mechanism of cold stress in rice pollen, providing a theoretical basis for guidance for inducing callus formation in rice pollen [72]. Arsenic (As) is a carcinogen, and its accumulation in rice can lead to growth restriction. To further investigate the potential mechanisms through which rice varieties respond to As stress, Ma et al., selected M2 and L5 as sensitive and tolerant varieties from 16 experimental varieties using a combination of transcriptomics and metabolomics approaches and integrated the results of transcriptomics and metabolomics to reveal the molecular mechanisms and processes associated with the responses of the two varieties to As stress [73]. By integrating transcriptomic and proteomic methods, Prathi et al. studied six rice genotypes, including two disease-tolerant varieties and four disease-susceptible varieties, to reveal the important role of DEPs in the rice variety *Rhizoctonia solani* [74]. Kuang et al., analyzed the root tips (RTs) and basal roots (BRs) of rice plants by integrating transcriptomic, proteomic, and metabolomic analyses and reported that cadmium (Cd) accumulation in RTs was 1.4 times greater than that in BRs, which revealed the different response mechanisms of rice RTs under Cd conditions. A molecular regulatory network, including genes, proteins, and metabolites in rice RTs was also proposed to cope with Cd toxicity, which is essential for ensuring rice food security [75].

## 4. Genomics-Based Innovations in Genetic Improvement Methods for Rice

As the global population continues to grow and economies grow, the need for food production and quality becomes more urgent. Against the backdrop of this challenge, increasing the yield, quality, and adaptability of rice, one of the world’s most important food crops, is critical. With the continuous development of high-throughput sequencing technologies and other biotechnologies, a series of novel genetic improvement methods, such as whole-genome navigation, molecular module design, breeding, and genomic selection (GS), have been developed and utilized. These advancements provide enormous potential for the promising future of rice breeding in academic research. Innovations in genetic improvement methods for rice can enhance breeding efficiency, reduce costs, and expedite genetic advancements, providing a more robust and sustainable foundation for agricultural production.

### 4.1. Genome-Wide Molecular Navigation Breeding

As a novel precision breeding method, molecular navigation breeding is increasingly emerging as a crucial tool for crop genetic improvement. Genome-wide molecular navigation involves the combination of molecular markers, quantitative trait nucleotide (QTN) allele frequency analysis, computer simulation, and machine learning to achieve efficient and precise methods for crop genetic improvement. First, information on the QTN functional variant loci of crops can be collected to reveal the correspondence between traits and genetic variants, and this information can be used to construct a key-functional-variant locus map. Second, with the help of advanced computer-simulation technology, the influence of different parameters on the screening probability can be evaluated effectively to optimize the breeding strategy. After determining the key parameters, these parameters can be substituted into the Molecular Navigation Breeding Simulation Program, and through scientific algorithms and precise calculations, the candidate materials for actual breeding will be screened out. Finally, to further improve the efficiency and accuracy of breeding, a genome navigation system (e.g., RiceNavi) can be used to optimize the breeding route.

Wei et al., studied eight genome-wide association-based research cohorts (QTNs), mapped rice quantitative trait nucleotides (QTNs) and inferred QTN effects, which illustrated that molecular markers can be used to resolve the genetic drag caused by uneven genome distribution when QTGs are mapped [76]. Wang et al., also conducted a comprehensive study integrating the genome, transcriptome, and population analysis of rice, identified key candidate genes for drought adaptation in upland rice (UR), and constructed a genomic navigation map for drought-resistant breeding in rice, revealing the crucial role of accumulated highland-specific variations in enhancing drought tolerance [77]. The application of molecular navigation breeding significantly enhances the efficiency of rice breeding, optimizes the cultivation of new varieties, and provides scientifically effective guidance for rice-breeding design.

### 4.2. Molecular Module Design Breeding

A molecular module typically refers to a functional unit composed of the main-effect genes and their interacting regulatory genes, which are responsible for achieving the expression of relevant functions and promoting the formation of complex target traits. Molecular module-design breeding is the theoretical simulation and functional prediction of analyzed molecular modules, enabling the combination of molecular modules to produce optimal nonlinear-additive effects, ultimately achieving targeted improvement of complex traits. Breeding through molecular module design can effectively address various extreme weather conditions, such as drought, high temperature, and salinization. Gu et al., demonstrated that the SGS3-tasiRNA-OsARF3 module is crucial for regulating heat tolerance and disease resistance in rice. This module positively regulates rice heat tolerance but negatively regulates rice immunity. These findings also indicate that such regulatory modules that are composed of multiple components may confer evolutionary advantages [78]. This comprehensive approach will provide novel ideas for global seed industry innovation, meeting the significant demands of global agriculture.

### 4.3. Genomic selection (GS)

Genomic selection (GS) is an innovative approach that utilizes genome-wide-DNA-marker data to improve the breeding efficiency of quantitative traits and predicts breeding value by comprehensively analyzing genomic data [79]. Compared to traditional phenotypic observation experiments, GS directly predicts the breeding value of complex phenotypes through phenotype-prediction models, greatly reducing cumbersome experimental steps and enabling efficient and rapid selection of materials. GS not only enables the prediction of multiple phenotypes using a single set of genomic data, thereby significantly reducing costs, but also enables breeders to accurately select breeding materials or design breeding schemes, ultimately shortening the breeding cycle. In the past, the major algorithms for GS included best linear unbiased prediction (BLUP) and Bayesian methods. The use of machine learning methods, especially deep learning methods, is growing rapidly; for example, a tool based on the LightGBM integrated model, called CropGBM, has been utilized for genomic selection for breeding. CropGBM stands out due to its outstanding model-training efficiency and feature selection capabilities [80,81]. Wang et al., enhanced the prediction accuracy of the relationship between plant genotype and phenotype by utilizing the deep neural network genomic prediction (DNNGP) deep learning method to predict plant genomic data [82].

Currently, research on rice genomic selection (GS) has focused mainly on designing training populations and evaluating predictive abilities within and between populations. Various quantitative traits in rice breeding populations have already undergone genomic prediction, demonstrating moderate to high predictive accuracy. Onogi et al. evaluated the predictive ability of nine methods, including genomic BLUP (GBLUP) and reproducible kernel Hilbert spatial regression (RKHS), by comparing Asian rice cultivar groups [83]. Zhang et al. utilized a population comprising 459 different rice varieties to assess factors influencing prediction and demonstrated that the reproducing kernel Hilbert space (RKHS) model exhibited the highest predictive ability. They further optimized the application of genomic selection by integrating genomic-wide association studies (GWAS) and various GS models [84]. GS can be utilized in hybrid rice breeding. For instance, Xu et al. demonstrated the utility of GS in rice breeding by comparing yield prediction values for the top 200 and bottom 200 grains among all potential hybrid combinations [85,86]. These studies provide a clear technical direction for big data-driven breeding. The direct guidance of genomic data for breeding practices can be achieved via GS, and the question of how to better optimize and utilize GS methods has become an important current breeding focus.

### 4.4. Quickly Improved Tetraploids

Compared with diploid plants, polyploid rice possesses advantages such as stronger stress resistance, disease and pest resistance, and greater adaptability. However, traditional polyploid rice tends to have a low seed setting rate. By conducting a series of studies on high-yielding and high-quality polyploid rice, new prospects for the quick improvement of rice have been revealed [87]. Yu et al., successfully identified a rice genotype (CCDD), named polyploid rice 1 (PPR1), by screening heterozygous tetraploid wild rice stocks, established an efficient transformation and genome-editing system, generated high-quality genome assemblies, and elucidated the genetic blueprint and evolutionary history of the heterozygous-tetraploid-rice genome. This study also describes the process of de novo domestication and improvement of a wild rice relative—the allotetraploid O. alta (CCDD). Additionally, genome-editing techniques were utilized to edit and enhance polyploid rice 1 (PPR1), which is associated with important agronomic traits in rice. This provides a pathway for achieving genetic improvement and breeding new staple grains [88].

### 4.5. Genome Editing

Genome-editing technology is an important tool for quickly obtaining breeding material with the intended genotype. The ZFN and TALEN systems are early systems used for genome editing. Shan et al. utilized TALEN technology to edit the fragrance gene BADH2 in rice, resulting in an increase in the aroma of ordinary rice [89]. In 2012, reliable and efficient genome-editing technology was first developed [90]. CRISPR–Cas originates from the bacterial immune system and is characterized by its high precision and efficiency. Its application in rice, fungi, and various plants holds the potential to propel agricultural research forward [91]. In 2013, researchers achieved gene editing in rice for the first time using CRISPR-Cas9 [92]. By knocking out the thermosensitive genic male sterility gene (TGMS) via the CRISPR-Cas9 system, Zhou et al., developed new male-sterile lines within a year [93]. Using genome-editing technology, breeders have developed rice varieties suitable for planting in cadmium-contaminated paddy fields [94]. Wang et al., successfully knocked out the functional alleles of the OsGhd7 gene using CRISPR-Cas9 technology, resulting in accelerated flowering in rice varieties. They demonstrated that modifying the functional alleles of OsGhd7 via CRISPR can lead to the breeding of early-maturing rice varieties [95]. Imran et al., employed CRISPR-Cas9 technology to edit the BADH2 gene, increasing the quality of the aroma of rice [96].

In 2016, researchers achieved the first instance of converting a specific cytosine (C) to thymine (T) at a particular genomic locus in human and murine cell lines when they completing single-base editing [97]. The following year, researchers successfully performed single-base editing of A·T-G·C in rice [98]. To enhance single-base editing efficiency, Zong et al. fused APOBEC3A (A3A-PBE) with a mutant form of Cas9, nCas9-D10A, enabling efficient single-base editing in rice [99]. In addition to Cas9, there are also new CRISPR systems available for genome editing. Hu et al. efficiently achieved genome editing in rice using the CRISPR-Gpf1 system, which requires only a single-stranded RNA composed of 42–44 nucleotides to recognize and cleave DNA. This system is more conducive to multi-gene editing, effectively expanding the application scope of genome-editing techniques in rice-genome editing [100,101]. With technological advancements, the rice genome will continue to be updated, and further exploration of rice genetics will provide more possibilities for future rice breeding and production.

## 5. Prospects of Genomics in Rice-Breeding Research

Early in rice genomics, the focus was on foundational research, which utilized systematic genome sequencing to unveil the structure and genetic diversity of the rice genome. In the intermediate stage, attention shifted toward functional gene exploration providing a deeper understanding of the biological functions of genes involved in rice growth, development, and responses to stress. With continuous innovations in genomic technologies, rice breeding has become more precise and efficient. The rice genome is relatively small but more than 3000 important functional genes have been reported, making it possible to create new varieties of rice more precisely. Future novel rice-breeding techniques will primarily revolve around whole-genome-breeding technology and genome-editing techniques. Rice is the first crop used for whole-genome-breeding technology, and novel molecular-marker techniques aim to achieve higher throughput, lower cost, and automated genetic screening by utilizing precise SNP arrays and gene typing based on resequencing [102]. High-throughput-genome-wide-molecular markers combined with superior gene screening have been widely used in rice breeding. Rice, which is conservatively used in the field of transgenic breeding, was the first crop used to practice gene editing in breeding, and as early as 2012, researchers edited the rice OsSWEET14 gene to obtain a white leaf blight-resistant line [103]. Gene editing offers several advantages over transgenic breeding, including the ability to edit multiple target sites and achieve precise edits at crucial gene loci. Additionally, gene editing allows for targeted insertion, replacement, and stacking of gene fragments in plants, making it a safer alternative to traditional transgenic methods. Rice has been used to achieve remarkable success in breeding for high yield, resistance, and quality traits, yet the full potential of the various rice species remains untapped. For instance, researchers have yet to identify effective genetic resources for high resistance to rice blight and rice blasts. In the future, with the aid of comprehensive rice-germplasm-genomic resources, researchers will be able to efficiently design varieties and continue to explore the breeding potential of rice using crop-phenomic analysis techniques, single-cell sequencing technologies, epigenomic analysis techniques, single-base editing, and gene replacement technologies.
